# *vcf2gwas*: Python API for comprehensive GWAS analysis using GEMMA

**DOI:** 10.1093/bioinformatics/btab710

**Published:** 2021-10-12

**Authors:** Frank Vogt, Gautam Shirsekar, Detlef Weigel

**Affiliations:** Department of Molecular Biology, Max Planck Institute for Developmental Biology, Tuebingen 72076, Germany; Department of Molecular Biology, Max Planck Institute for Developmental Biology, Tuebingen 72076, Germany; Department of Molecular Biology, Max Planck Institute for Developmental Biology, Tuebingen 72076, Germany

## Abstract

**Motivation:**

Genome-wide association study (GWAS) requires a researcher to perform a multitude of different actions during analysis. From editing and formatting genotype and phenotype information to running the analysis software to summarizing and visualizing the results. A typical GWAS workflow poses a significant challenge of utilizing the command-line, manual text-editing and requiring knowledge of one or more programming/scripting languages, especially for newcomers.

**Results:**

*vcf2gwas* is a package that provides a convenient pipeline to perform all of the steps of a traditional GWAS workflow by reducing it to a single command-line input of a Variant Call Format file and a phenotype data file. In addition, all the required software is installed with the package. *vcf2gwas* also implements several useful features enhancing the reproducibility of GWAS analysis.

**Availability and implementation:**

The source code of *vcf2gwas* is available under the GNU General Public License. The package can be easily installed using conda. Installation instructions and a manual including tutorials can be accessed on the package website at https://github.com/frankvogt/vcf2gwas.

**Supplementary information:**

Supplementary data are available at *Bioinformatics* online.

## 1 Introduction

Genome-wide association study (GWAS) has been proven to be an extremely useful tool to find an association between genetic variants, typically single-nucleotide polymorphisms (SNPs) and a given trait in many organisms. GWAS needs information of traits of a set of individuals under investigation (phenotypes) and genotypes (SNPs) of these individuals obtained through DNA sequencing. During the analysis, genetic variants are tested for the association with the phenotypes using various computational methods implemented in a wide range of software (Uffelmann *et al.*, 2021). The likelihood of each SNP to be associated with the trait is calculated and subsequently used to identify SNPs with significant associations.

While there are many algorithms implemented in various software to perform the association analysis, Genome-wide Efficient Mixed Model Association (GEMMA) (Zhou and Stephens, 2012) stands out because of its versatility and efficiency in handling large-scale data. GEMMA can fit a univariate linear mixed model (Zhou and Stephens, 2012), a multivariate mixed model (Zhou and Stephens, 2014) and a Bayesian sparse linear mixed model (Zhou *et al.*, 2013) for testing marker associations with a trait of interest in different organisms.

Although GEMMA has a very straightforward command-line interface to carry out the actual association analysis, it requires users to first install necessary software with required dependencies on their machines. Subsequently, users need to prepare the inputs (genotype and phenotype) in a proper format before execution of GEMMA. Similarly, the outputs generated need post-processing of the results for better interpretation and presentation. The entire workflow can be overwhelming especially for inexperienced users. Briefly, this workflow starts with the genotype information in a Variant Call Format (VCF) file. This file has to be converted to the PLINK (Purcell *et al.*, 2007) BED format with the phenotype information that needs to be manually edited into the associated .FAM file. Once the analysis is complete, the user is left with the outputs which need to be summarized and plotted. If multiple phenotypes are to be analyzed, the analysis has to be repeated for every phenotype. Thus, performing GWAS in this fashion using GEMMA can be time-consuming and makes it challenging to keep the analyses well-organized to enhance reproducibility.

The *vcf2gwas* package aims to facilitate performing a GWAS with GEMMA by automating all the phases, beginning with the installation of all the required software, to input preparations, to carrying out the analysis, and finally to processing the results. *vcf2gwas* avoids steps such as setting up a configuration file that are common in Nextflow, Snakemake-based pipelines, and also avoids commonly experienced issues with root privileges necessary for running an application inside a Docker container. Thus, these features allow novice users to focus on the analysis rather than technical aspects of installation and execution. *vcf2gwas* is especially helpful when analyzing large numbers of phenotypes or different sets of individuals because it can perform the analyses in parallel with a single.csv file with all the phenotypes. In addition, the package offers features like analyzing reduced phenotypic space and comparing the SNPs showing significant association to genes or regions of interest. *vcf2gwas* is easily installable as a conda package and therefore makes it possible to reproduce each GWAS on any compatible machine. All these user-friendly features of *vcf2gwas* make GWAS analysis easily accessible across various diploid model and non-model organisms.

## 2 Implementation

The *vcf2gwas* package is implemented using the Python programming language. It contains multiple Python3 scripts with all the functions required for the program to execute the analysis. The core functionality of the package consists of the ability to perform automated GWAS analysis with a single command-line input, requiring minimal overhead by the user. It has multiple wrapper functions calling bcftools (Danecek *et al.*, 2021), PLINK (Purcell *et al.*, 2007) and GEMMA (Zhou and Stephens, 2012) from Python3 as well as complementary Python3 functions performing both pre- and post-analysis tasks, all executed by the pipeline scripts. Pre-analysis includes functions for formatting, trimming and filtering the input files for the association analysis with GEMMA. The different analysis modes of GEMMA can be utilized and are also specified with the command-line input. In addition, covariates can also be included in the association analysis performed with either linear model or linear mixed model. For the post-analysis, additional functions are used to summarize and visualize the resulting data and perform any extra operations specified by the command-line options. A tree-like directory structure is generated to save all the results associated with the analysis of a given phenotype. *vcf2gwas* uses Python libraries numpy, matplotlib, pandas for preparing input/output files and plotting, while scikit-learn and umap-learn libraries are used for dimensionality reduction (details of the library versions can be found in the repository at https://www.github.com/frankvogt/vcf2gwas). *vcf2gwas* has been successfully tested on machines running a Unix-based OS (macOS/Linux).

## 3 Example usage and performance

*vcf2gwas* trims, filters and converts input VCF file and formats the phenotype file as required by GEMMA. A basic bash command example to analyze a single phenotype using GEMMA’s linear mixed model providing only the VCF and phenotype file (in the csv format) is:

$vcf2gwas−v<input.vcf>−pf<inputpheno.csv>−p1−lmm

**Fig. 1.**
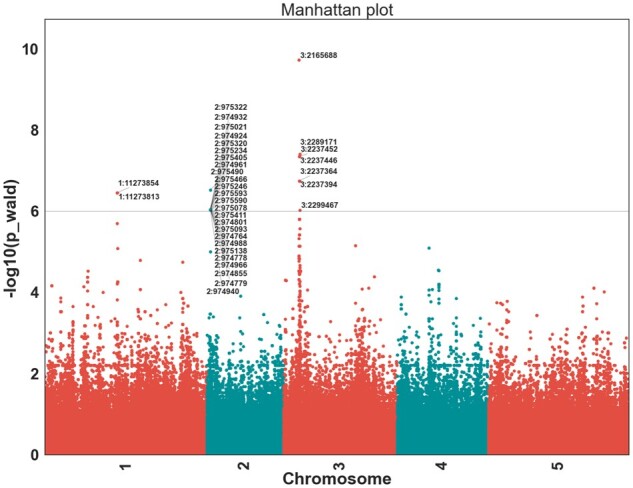
Manhattan plot produced by *vcf2gwas* on *avrRpm1* recognition in *A.thaliana* (The nearest significant SNP to *A.thaliana Rpm1* resistance gene on chromosome 3 is 8340 bp upstream.)

We performed GWAS analysis on a hypersensitive response phenotype observed in 58 *Arabidopsis* *thaliana* host lines (∼900 000 SNPs) when infected with *Pseudomonas syringe* expressing *avrRpm1* gene using the linear mixed model (lmm). Description of the original experiment can be found at https://arapheno.1001genomes.org/phenotype/17/. After the analysis is executed, the results are summarized and visualized with a publication-ready Manhattan plot (Fig. 1) and Q–Q plot (Supplementary Fig. S1). 

*vcf2gwas* was benchmarked by analyzing the same dataset with linkage disequilibrium (LD)-based pruning of the SNPs at different *R*^2^ thresholds. The wall-clock time and the number of significant SNPs were compared against the number of total SNPs retained after LD-pruning, independently. The results are shown in Supplementary Figures S2 and S3. In addition, we compared *vcf2gwas* with other available GWAS workflows. The comparison is presented in Supplementary Table S2.

## 4 Additional functionality

Beyond summarizing and plotting the results of the GEMMA analysis, following important additional features are implemented in *vcf2gwas* (for a complete list of features, see Supplementary Table S1):

- Dimensionality reduction via PCA or UMAP can be performed on phenotypes and used for analysis.
- Relevant SNPs can be compared with genes/regions of interest listed in additionally specified file (see Supplementary Table S3)
- *vcf2gwas* is able to analyze several input files with different sets of individuals and multiple phenotypes in an efficient manner due to parallelization, saving the user a lot of time compared with standard GWAS procedure.

## Supplementary Material

btab710_Supplementary_Data
