# Supplementary material for: vcf2gwas: Python API for comprehensive GWAS analysis using GEMMA
Source: Bioinformatics. 2021 Oct 12;38(3):839–40. doi: 10.1093/bioinformatics/btab710 (PMC8756188; doi:10.1093/bioinformatics/btab710)
Supplement: btab710_Supplementary_Data [file btab710_supplementary_data.pdf]

Supplementary Figures

List of Supplementary Figures

1 Q-Q plot produced by *vcf2gwas* on association analysis performed on the *avrRpm1* recognition in *Arabidopsis thaliana* using the linear mixed model. 2
2 *vcf2gwas* runtime as a function of different level of linkage disequilibrium (LD)-based pruning of the original 1001 Genomes SNP dataset. As in Figure S 1, the *avrRpm1* phenotype and the linear mixed model were used for all runs. 2
3 Number of significant SNPs as a function of SNPs retained by different LD-pruning thresholds. As in Figure S 1, the *avrRpm1* phenotype and the linear mixed model were used for all runs. 5

Supplementary Tables

List of Supplementary Tables

1 List of *vcf2gwas*'s most important features, listed separately by the main phases of a GWAS. An extensive list of every command-line option is available in the manual (`https://github.com/frankvogt/vcf2gwas/blob/main/MANUAL.md`). 3
2 List of features and characteristics of *vcf2gwas* compared to two often used GWAS pipelines, *Hail* (`https://hail.is/`) and *easyGWAS* (`https://easygwas.ethz.ch/`). More information on the dataset used to compare the runtime can be found at `https://github.com/frankvogt/vcf2gwas/tree/main/files/Comparison` 4
3 List of SNPs above the significance threshold ($-log_{10}(10^{-7})$) (using the same dataset as in Figure 1 and Figure S 1) and their distance from a given gene set (here, *Arabidopsis thaliana* immune system genes containing NBS-LRR domain) produced by *vcf2gwas*, indicating possible relevance of certain genes to the analyzed phenotype for which association analysis was performed. 6

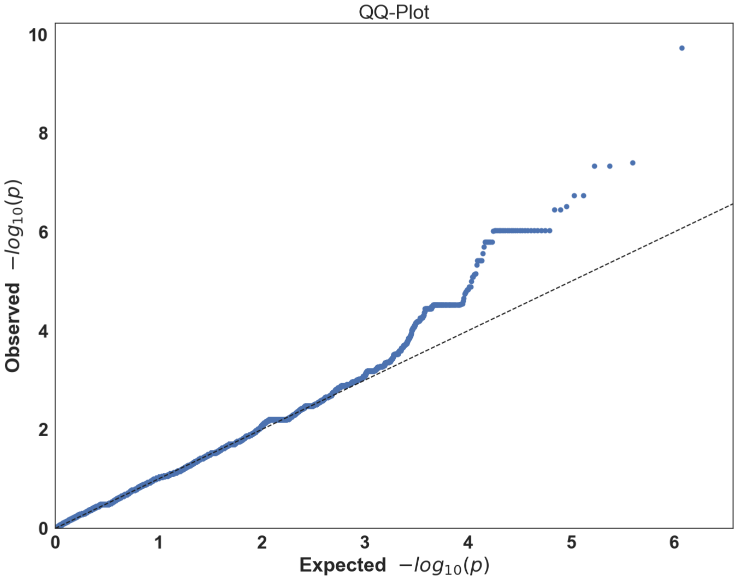

Figure S 1: Q-Q plot produced by *vcf2gwas* on association analysis performed on the *avrRpm1* recognition in *Arabidopsis thaliana* using the linear mixed model.

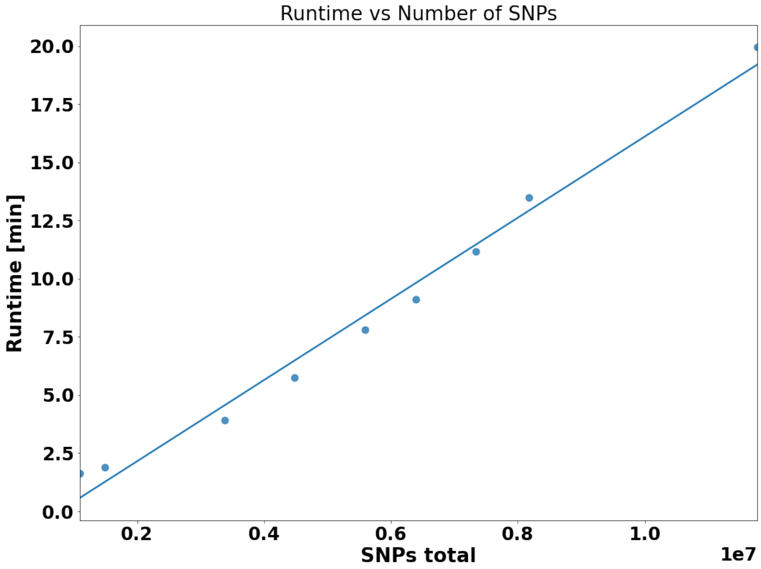

Figure S 2: *vcf2gwas* runtime as a function of different level of linkage disequilibrium (LD)-based pruning of the original 1001 Genomes SNP dataset. As in Figure S 1, the *avrRpm1* phenotype and the linear mixed model were used for all runs.

Area	Main features
Installation	Installation via Conda, dependencies (bcftools, plink, and GEMMA) are installed automatically
Input files	No pre-formatting for VCF file required, CSV file for phenotypes/covariates,
File preparations	Individuals in all files are adjusted & ordered, correctly formatted for GEMMA analysis
Analysis	Full access to GEMMA's algorithms (LM, LMM, mvLMM, BSLMM)
Output	• Quality control plots for phenotype and genotype data<br>• Q-Q plots, Manhattan plots, diagnostic plots for BSLMM, SNP output tables
Summary	• Summary tables (especially useful when analyzing multiple phenotypes)<br>• Comparing top SNPs to genes of interest (gene files of common species built-in or via GFF/CSV input file)
Additional features	• Analysis of synthetic phenotypes using dimensionality reduction via PCA or UMAP<br>• Principal components (PCs) of genotype data can be extracted & used as covariates<br>• PCs of genotype data can used instead of GEMMA's standard relatedness matrix<br>• Efficiency due to parallelization when analyzing multiple phenotypes from one or more phenotype files at the same time<br>• Analysis and outputs customizable with various additional options<br>• Results saved in clear hierarchical directory structure

Table S 1: List of *vcf2gwas*'s most important features, listed separately by the main phases of a GWAS. An extensive list of every command-line option is available in the manual (`https://github.com/frankvogt/vcf2gwas/blob/main/MANUAL.md`).

	**vcf2gwas**	**Hail**	**easyGWAS**
Platforms	macOS, Linux	macOS, Linux	online (web-based)
Installation	via Conda	via pip	no installation necessary
Usage	Command line	Jupyter notebook based	web-based interface
Genotype input format	VCF	VCF, PLINK BED, PLINK FAM etc.	PLINK PED
Phenotype input format	CSV	CSV/TSV, JSON, FAM etc.	TSV (PLINK format)
Pre-formatting required (from vcf file)	No	Yes	Yes
Plots	Yes	Yes	Yes
Plots saved	by default	manually	downloadable
Summary files saved	by default	manually	downloadable
Quality control	Yes	Yes	Yes
Filtering (minor allele frequency)	Yes	Yes	Yes
Comparison to genes of interest	Yes	Yes	Yes
Genotype dimension reduction	Yes (PCA)	Yes (PCA)	Yes (PCA)
Phenotype dimension reduction	Yes (PCA, UMAP)	No	No
Phenotypes per analysis	arbitrary	arbitrary	maximum 5
Parallel analysis of multiple phenotypes	Yes	Yes	No (separate analysis for each phenotype)
Algorithm	GEMMA (LM, LMM, mvLMM, BSLMM)	LMM, linear/logistic/poisson regression	EMMAX, linear regression, FaSTLMM
Runtime (∼12,000 SNPs, 19 chromosomes, 1940 individuals)	∼5 min	∼30 s	∼3 min

Table S 2: List of features and characteristics of *vcf2gwas* compared to two often used GWAS pipelines, *Hail* (`https://hail.is/`) and *easyGWAS* (`https://easygwas.ethz.ch/`). More information on the dataset used to compare the runtime can be found at `https://github.com/frankvogt/vcf2gwas/tree/main/files/Comparison`

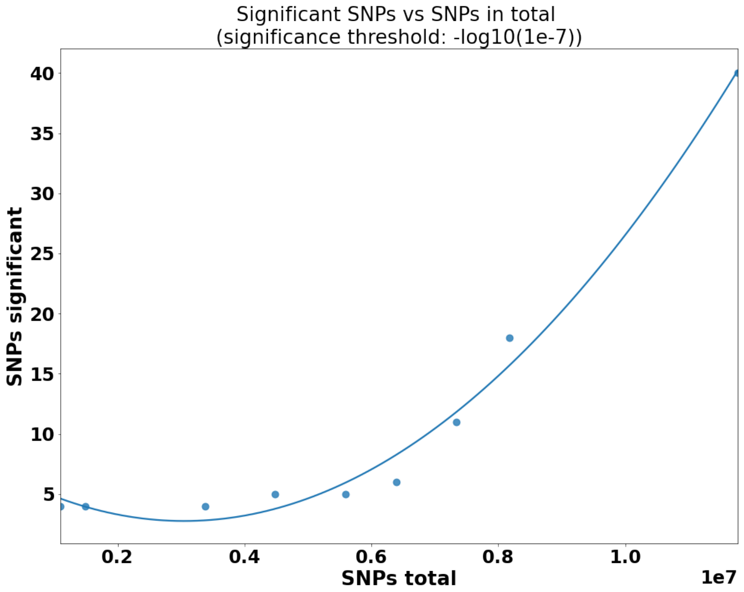

Figure S 3: Number of significant SNPs as a function of SNPs retained by different LD-pruning thresholds. As in Figure S 1, the *avrRpm1* phenotype and the linear mixed model were used for all runs.

The threshold to distinguish significant SNPs is $-log_{10}(10^{-7})$.

SNP ID	Chr	Phenotype	Upstream gene				SNP position	Downstream gene			
			ID	Comment	Name	Distance		Distance	Name	Comment	ID
3:2237364	3	avrRpm	AT3G07040.1	NB-ARC domain-containing disease resistance protein	RPM1	8340	2237364				
3:2237394	3	avrRpm	AT3G07040.1	NB-ARC domain-containing disease resistance protein	RPM1	8370	2237394				
3:2237446	3	avrRpm	AT3G07040.1	NB-ARC domain-containing disease resistance protein	RPM1	8422	2237446				
3:2237452	3	avrRpm	AT3G07040.1	NB-ARC domain-containing disease resistance protein	RPM1	8428	2237452				
3:2289171	3	avrRpm	AT3G07040.1	NB-ARC domain-containing disease resistance protein	RPM1	60147	2289171				
2:975138	2	avrRpm	AT2G03030.1	Toll-Interleukin-Resistance (TIR) domain family protein		84618	975138	28330		Toll-Interleukin-Resistance (TIR) domain family protein	AT2G03300.1
2:975234	2	avrRpm	AT2G03030.1	Toll-Interleukin-Resistance (TIR) domain family protein		84714	975234	28234		Toll-Interleukin-Resistance (TIR) domain family protein	AT2G03300.1
2:975320	2	avrRpm	AT2G03030.1	Toll-Interleukin-Resistance (TIR) domain family protein		84800	975320	28148		Toll-Interleukin-Resistance (TIR) domain family protein	AT2G03300.1
2:975405	2	avrRpm	AT2G03030.1	Toll-Interleukin-Resistance (TIR) domain family protein		84885	975405	28063		Toll-Interleukin-Resistance (TIR) domain family protein	AT2G03300.1
2:975411	2	avrRpm	AT2G03030.1	Toll-Interleukin-Resistance (TIR) domain family protein		84891	975411	28057		Toll-Interleukin-Resistance (TIR) domain family protein	AT2G03300.1
2:975490	2	avrRpm	AT2G03030.1	Toll-Interleukin-Resistance (TIR) domain family protein		84970	975490	27978		Toll-Interleukin-Resistance (TIR) domain family protein	AT2G03300.1
2:975590	2	avrRpm	AT2G03030.1	Toll-Interleukin-Resistance (TIR) domain family protein		85070	975590	27878		Toll-Interleukin-Resistance (TIR) domain family protein	AT2G03300.1
1:11273854	1	avrRpm					11273854	14598	RAC1	Disease resistance protein (TIR-NBS-LRR class) family	AT1G31540.2
1:11273813	1	avrRpm					11273813	14639	RAC1	Disease resistance protein (TIR-NBS-LRR class) family	AT1G31540.2
3:2165688	3	avrRpm					2165688	60264	RPM1	NB-ARC domain-containing disease resistance protein	AT3G07040.1

Table S 3: List of SNPs above the significance threshold ($-log_{10}(10^{-7})$) (using the same dataset as in Figure 1 and Figure S 1) and their distance from a given gene set (here, *Arabidopsis thaliana* immune system genes containing NBS-LRR domain) produced by *vcf2gwas*, indicating possible relevance of certain genes to the analyzed phenotype for which association analysis was performed.
